# 423. Implementation of a Hierarchy of Controls in a Mobile Health Unit to Safely Care for Inpatients with COVID-19 during Healthcare System Surge

**DOI:** 10.1093/ofid/ofab466.623

**Published:** 2021-12-04

**Authors:** Paige Reason, Jerome A Leis, Claudia Cocco, Lynfa Stroud, Michelle Hladunewich, Debra Carew, Robert Burgess, Christina Chan, Victoria R Williams, Nicholas Tomiczek, Natasha Salt, Adrienne Chan

**Affiliations:** Sunnybrook Health Sciences Centre, Toronto, Ontario, Canada

## Abstract

**Background:**

In April 2021, Sunnybrook Health Sciences Centre opened a Mobile Health Unit (MHU, i.e. medical tents) under the direction of the Ontario Ministry of Health and Long Term Care in response to a surge in hospitalized patients with COVID-19 during wave three of the pandemic. Providing care to patients in non-conventional spaces is not new, however, experience in safely caring for COVID-19 patients in these settings is lacking. Our aim is to describe the implementation of our MHU and associated outcomes of these COVID-19 patients.

**Methods:**

A multidisciplinary clinical and operations team was created to plan, execute and operate a safe environment for COVID-19 patients and healthcare workers within the MHU. Patient selection was restricted to patients with COVID-19 who were clinically recovering from severe COVID-19 pneumonia. Ventilation was optimized with air flow directed away from patient areas, velocity reduced to below 0.25 meters per second, and air exchanges of 24-28 per hour. All healthcare workers working in the MHU were offered COVID-19 vaccine and required to complete mandatory education if they declined (vaccination rate of 87% was achieved among dedicated staff). Universal masking and eye protection was used throughout the MHU with designated areas for donning and doffing personal protective equipment.

**Results:**

In total, 32 patients with COVID-19 were managed in the MHU between 26 April and 21 May, 2021. Table 1 provides the summary of patient characteristics. All patients had a median of one-day of transmission-based precautions remaining in their course and were infected with Alpha variant with exception of one patient with the Gamma variant. Among those patients with genotyping available, all were infected with SARS-CoV-2 carrying the N501Y mutation. Four of the 32 patients required transfer to the main hospital for medical indication while the others were discharged home or to rehabilitation. None of the healthcare workers who worked within the MHU developed COVID-19 infection.

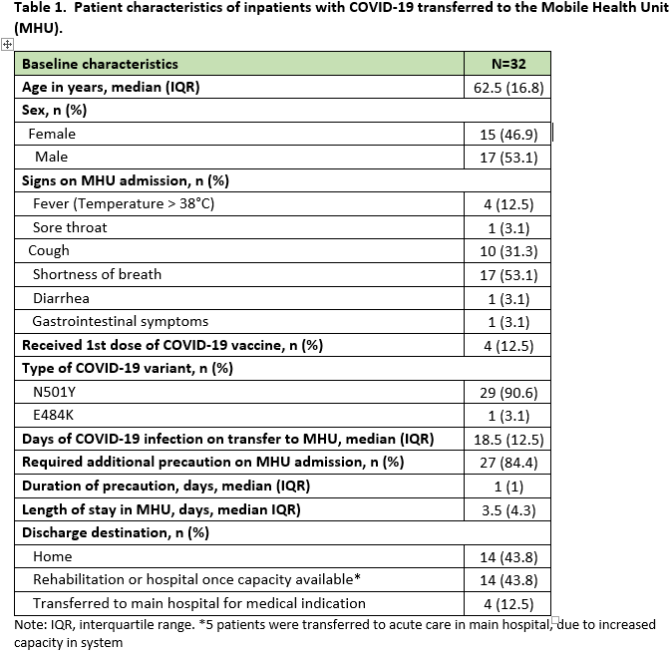

**Conclusion:**

We safely cared for patients recovering from COVID-19 infection in an MHU to support system healthcare capacity. Our experience, including the specific hierarchy of controls implemented, may be helpful for future pandemic planning.

**Disclosures:**

**All Authors**: No reported disclosures

